# Imaging of the sublingual and submandibular spaces

**DOI:** 10.1007/s13244-018-0615-4

**Published:** 2018-04-19

**Authors:** Swapnil Patel, Alok A. Bhatt

**Affiliations:** Department of Imaging Sciences, University of Rochester Medical Center, 601 Elmwood Avenue, P.O. Box 648, Rochester, NY 14642 USA

**Keywords:** Head and neck, Salivary glands, Education, CT, MR

## Abstract

**Abstract:**

Divided by the mylohyoid muscle, the sublingual and submandibular spaces represent a relatively small part of the oral cavity, but account for a disproportionate amount of pathological processes. These entities are traditionally separated into congenital, infectious/inflammatory, vascular and neoplastic aetiologies. This article reviews the relevant anatomy, clinical highlights and distinguishing imaging features necessary for accurate characterisation.

**Teaching Points:**

• *The mylohyoid sling is a key anatomical landmark useful in surgical planning.*

• *Congenital lesions and infectious/inflammatory processes constitute the majority of pathology.*

• *Depth of invasion is key when staging tumours in the oral cavity.*

## Introduction

The suprahyoid neck is frequently the site of many common conditions. Although accessible to clinical exam, some components are better evaluated with imaging; lesions occupying the sublingual space and submandibular space are invisible to the referring clinician. A broad array of pathological processes can occur in these spaces and pinpointing the origin can narrow the differential and in certain cases determine the diagnosis. These lesions can be classified into congenital, infectious/inflammatory, vascular and neoplastic processes. Typically, computed tomography (CT) or magnetic resonance imaging (MRI) are used for evaluation, but there may be cases where both are needed in conjunction. This review article discusses the relevant anatomy, clinical highlights and the characteristic imaging features of the various pathologies that occur within the sublingual and submandibular spaces.

## Embryology/anatomy

Within the suprahyoid neck, the sublingual and submandibular spaces are the site of a large variety of pathology, given their embryological origin and contents [1].

**Fig. 1:**
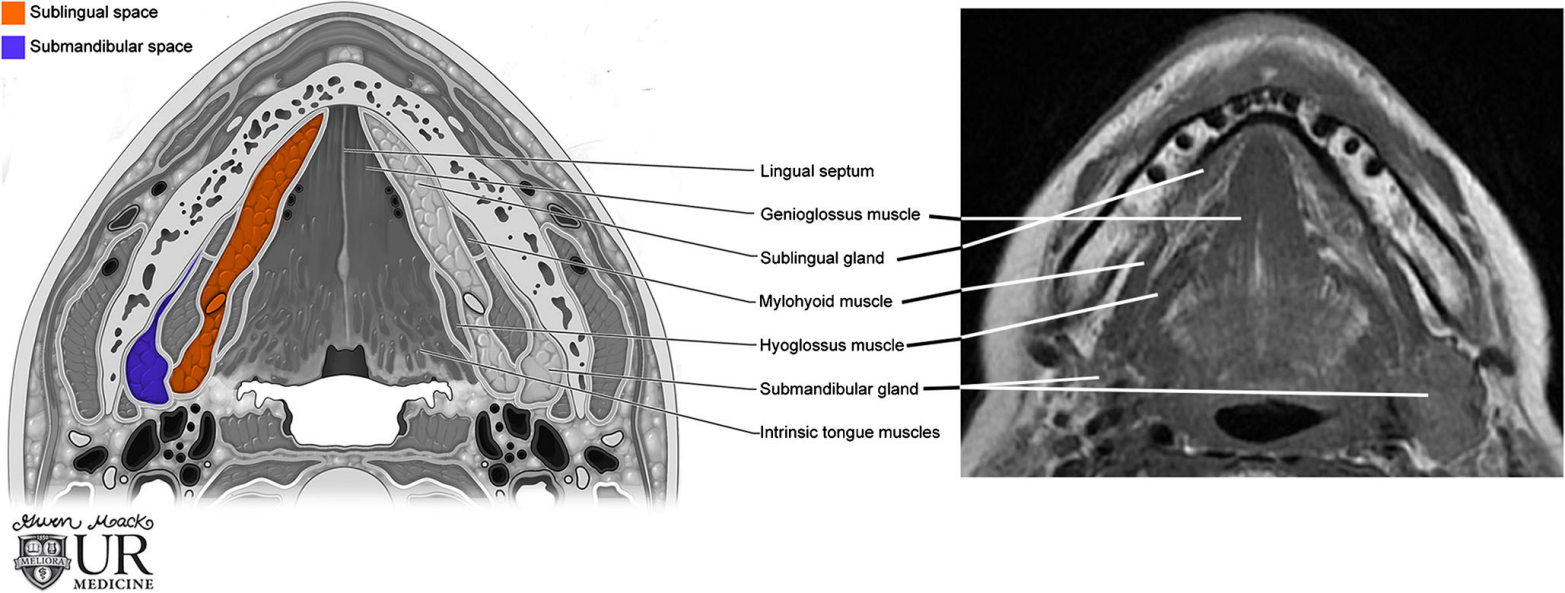
Anatomy of the submandibular and sublingual spaces in the axial plane: picture illustration and T1-weighted MR image

**Fig. 2:**
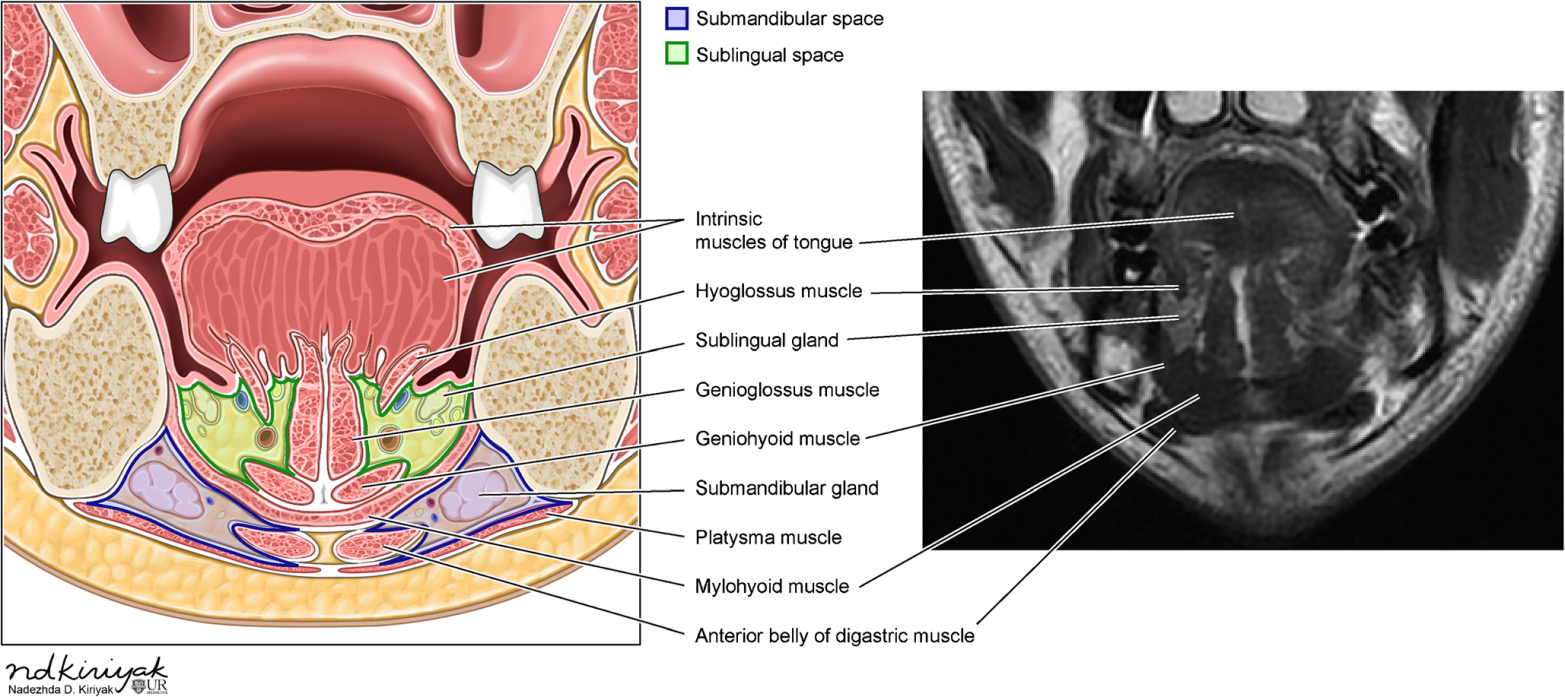
Anatomy of the submandibular and sublingual spaces in the coronal plane: picture illustration and T2-weighted MR image

The sublingual space is bounded anteriorly by the mandible, medially by the midline genioglossus/geniohyoid muscle complex, inferolaterally by the mylohyoid muscle, and superomedially by the mucosa of the floor of the mouth and intrinsic tongue muscles (Figs. 1 and 2). Communication of the right and left sublingual space occurs anteriorly under the frenulum of the tongue [1].

Although predominantly fat-containing, the sublingual space also contains the sublingual gland and duct, a portion of the hyoglossus muscle, the lingual artery and vein, lingual nerve (branch of CN V), branches of the glossopharyngeal (CN IX) and hypoglossal (CN XII) nerves, as well as the deep portion of the submandibular gland and duct [2].

The submandibular space is bounded anteriorly and laterally by the mandible, medially by the anterior belly of the digastric muscles, superiorly by the mylohyoid muscle and inferiorly by the hyoid bone (Figs. 1 and 2). The space is enclosed by the superficial layer of the deep cervical fascia, except along the posterior margin of the mylohyoid muscle, permitting continuity with the sublingual space and potential communication with the parapharyngeal space through a buccopharyngeal gap created by the styloglossus muscle. The primary contents include the superficial portion of the submandibular gland, submandibular lymph nodes and fat. The facial artery and vein, as well as a portion of the hypoglossal nerve, course through the space [2].

## Pathology

A variety of disease processes occur in the sublingual and submandibular spaces, and can be broadly classified into congenital, infectious, vascular and neoplastic aetiologies (Table 1). CT and MRI are the modalities of choice for imaging this region; CT is readily available, and MRI provides superior soft tissue resolution. Other commonly used modalities such as ultrasound, conventional sialography and positron emission tomography (PET) will be presented in order to illustrate findings on different modalities; often, pathology of these spaces may be discovered on exams performed for other reasons.

**Table 1:** Categorisation of spectrum of pathology

Congenital	Vascular	Infection/inflammation	Neoplasm
Aplasia/hypoplasia of salivary glands	Low flow	Cellulitis	Benign
	-Venous malformations	Ludwig angina	-Lipoma
Dermoid/Epidermoid cyst	-Lymphatic malformations	Abscess Ranula	-Primary salivary gland tumours (ie. pleomorphic adneoma) -Neurogenic tumours
	High flow		Malignant
	-Arterial malformations	Sialoadenitis	-Squamous cell carcinoma -Primary salivary gland tumours (ie. mucoepidermoid carcinoma) -Lymphoma

## Congenital

### Aplasia/hypoplasia of salivary glands

Aplasia of the salivary glands is a rare condition that usually affects the parotid and submandibular glands. Single or multiple glands may be absent or hypoplastic. Exact aetiology remains unknown; however, the condition has been attributed to first and second branchial arch abnormalities. Specifically, it has been associated with genetic syndromes involving craniofacial development such as Treacher-Collins syndrome, hemifacial microsomia and lacrimo-auriculo-dento-digital syndrome [3]. Some studies report increased incidence in trisomy 21 [4]. Although usually discovered incidentally on imaging, some cases are identified when patients present with dry mouth, dysphagia and dental disease. Imaging may show compensatory hypertrophy of other major salivary glands, and therefore it is important not to mistake normal gland tissue for other lesions such as tumour [5].

### Dermoid and epidermoid cysts

True dermoid cysts (also known as benign cystic teratomas), epidermoid cysts and teratoid cysts represent a spectrum of congenital and acquired cystic malformations sharing the common characteristic of a squamous epithelial lining. Dermoid cysts as a term refers to three histologically distinct processes classified based on whether they are lined with simple squamous epithelium (epidermoid), skin appendages (dermoid) or tissues of other major organs (teratoid). Dermoids and epidermoids arise from dermal elements of the first and second branchial arches [1, 6]. It is important to distinguish dermoid and epidermoid cysts apart from teratoid cysts, as the former harbour a lower risk of malignant degeneration [7]. Although rare within the head and neck, dermoid and epidermoid cysts have a predilection for the oral cavity, specifically the floor of the mouth [8]. Dermoid cysts typically present in the 2nd or 3rd decade of life as a slowly enlarging midline neck mass, which may progress to cause dysphagia, while epidermoid cysts manifest earlier in life, becoming evident in infancy.

**Fig. 3:**
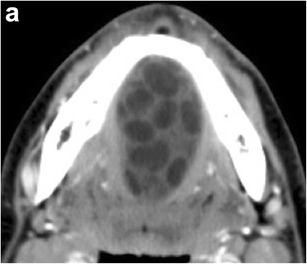
Dermoid cyst. Axial contrast-enhanced CT image shows a lesion with globules of fat (“sac of marbles”) in a fluid medium splaying the genioglossus muscles laterally to their respective ipsilateral sides

**Fig. 4:**
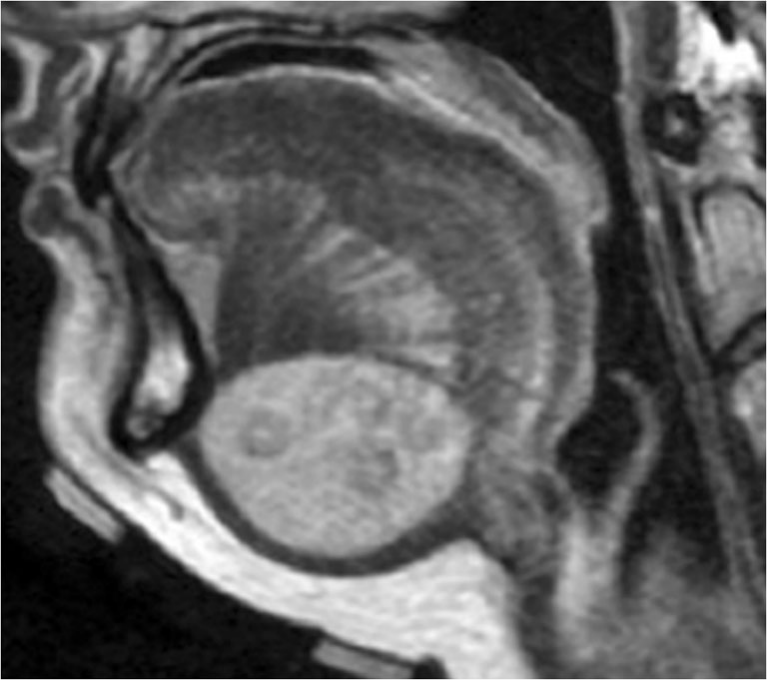
Dermoid cyst. Sagittal T2-weighted MR image shows a well-circumscribed cystic lesion filled with fat globules (“sac of marbles”)

On imaging, dermoid and epidermoid cysts can have a similar appearance. Some of the distinguishing features are derived from their unique histological composition. While both behave as cystic lesions on CT and MRI, dermoid cysts will demonstrate the hallmark finding of coalesced globules of fat resembling a “sac of marbles” in a medium (mixture of accessory dermal contents such as sebum and calcification) of low CT attenuation and variable signal on T1-weighted images (Figs. 3 and 4) [9]. Epidermoid cysts will distinctively show diffusion restriction on MRI [10]. The relationship of these cystic lesions to the mylohyoid muscle is important in guiding surgical intervention, as cysts superior to the mylohyoid muscle are removed preferably with an intraoral approach, while inferior lesions are excised through an external submandibular approach [11].

## Infectious/inflammatory

There are many causes, as well sequelae of inflammation and infection within the sublingual and submandibular spaces. Proper localisation of the abnormality is useful in determining aetiology.

### Ranula

Among the differential diagnosis of cystic lesions in the sublingual space, a ranula is a commonly acquired lesion (Table 2). Traditionally considered as a post-inflammatory or post-traumatic sequela of glandular obstruction, a ranula is a mucous retention cyst arising from the sublingual or minor salivary glands [12].

**Table 2:** Distinguishing imaging features among cystic lesions in the sublingual and submandibular spaces

	Ranula	Dermoid	Epidermoid
CT	-Well circumscribed cyst	-Mixed cystic and solid -May contain calcifications	-Well circumscribed cyst -No distinct solid component
MR	-Fluid signal with variable T1 signal (proteinaceous contents)	-Fat globules with “sack of marbles” appearance -Heterogenous signal due to dermal elements and cystic composition	-Diffusion restriction
Site	-Typically associated with the salivary glands, commonly sublingual gland -May extend into submandibular space (plunging ranula)	-Most commonly in the floor of the mouth, specifically within the sublingual and submandibular spaces	-Most commonly in the floor of the mouth
Histology	-Resemble mucous retention cyst	-Presence of skin appendages (hair, sebaceous glands, sweat glands)	-Lack of skin appendages

**Fig. 5:**
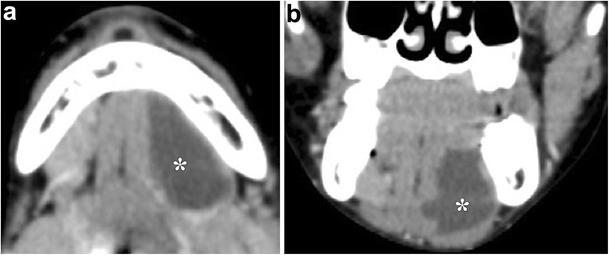
Simple ranula. **a** Axial and **b** coronal contrast-enhanced CT images demonstrate a well-circumscribed fluid attenuation lesion within the left sublingual space (*)

**Fig. 6:**
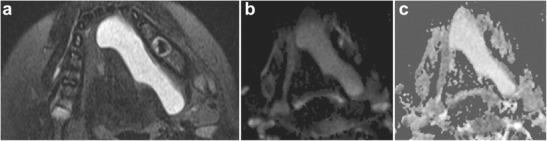
Sublingual space ranula. **a** Axial T2-weighted MR image shows a high-signal lesion within the left sublingual space. **b** Axial diffusion-weighted image and **c** ADC map demonstrates lack of restricted diffusion, confirming the diagnosis (as opposed to an epidermoid, which would have restricted diffusion)

On imaging, ranulas have the typical appearance of cystic lesions with fluid attenuation on CT, hyperintensity on T2-weighted MR sequences, and are anechoic on ultrasound (Fig. 5). MRI in particular can serve to assess the size, content, location and extent of the lesion, especially in preoperative planning [13]. Additionally, a ranula can be indistinguishable from an epidermoid cyst based on CT and sonography, but the absence of restricted diffusion on MRI establishes the diagnosis (Fig. 6) [14].

**Fig. 7:**
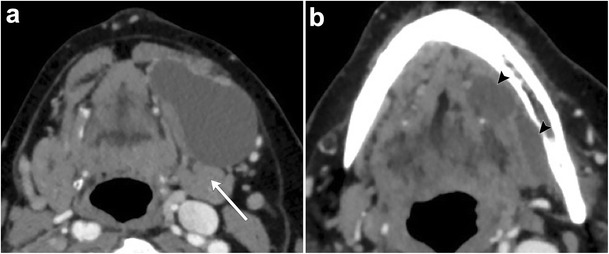
Plunging ranula. Axial contrast-enhanced CT images of the floor of the mouth illustrates a well-circumscribed cystic lesion predominantly occupying the left submandibular space causing a mass effect on the left submandibular gland (**a**, *arrow*). A more superior image reveals a small portion of the lesion based in the sublingual space, referred to as the “tail sign” (**b**, *arrowheads*)

As the cystic lesion enlarges, it can “rupture” through its sublingual space boundaries and extend into the submandibular space through the posterior free edge of the mylohyoid muscle or mylohyoid boutonniere. Referred to as a plunging ranula, the herniated portion leaves behind a band-like portion of the cyst within the sublingual space, forming the “tail sign” (Fig. 7) [15]. On rare occasions, it can also extend into the parapharyngeal space or upper cervical soft tissues [16].

### Cellulitis/abscess

Cellulitis is a diffuse infectious process of the skin and subcutaneous tissues. In the oral cavity, infections typically arise from a glandular or dental aetiology and may be limited to a single compartment or be a trans-spatial process [17]. Most commonly diagnosed with CT, key findings include skin thickening, fat stranding and enhancement of fascial planes [18].

**Fig. 8:**
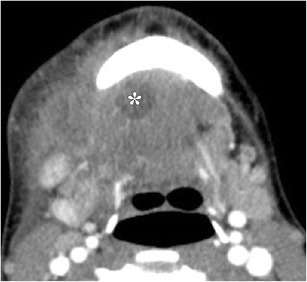
Sublingual/submandibular space abscess. Axial contrast-enhanced CT image through the floor of the mouth demonstrates extensive subcutaneous fat stranding and inflammatory changes centred within the right submandibular and sublingual spaces. A well-circumscribed fluid-attenuating collection is present within the right sublingual space consistent with early abscess formation (*)

As infection progresses unimpeded, bands of fluid coalesce into well-defined rim-enhancing collections or abscesses (Fig. 8). Route of spread in dental infection is dictated by fascial planes and muscle attachments. In particular, the posterior attachment of the mylohyoid muscle is at the level of the third mandibular molar. Infections originating in the apex of posterior mandibular teeth (i.e. third molar) are inferior to the mylohyoid insertion and tend to involve the submandibular space [13].

### Ludwig angina

**Fig. 9:**
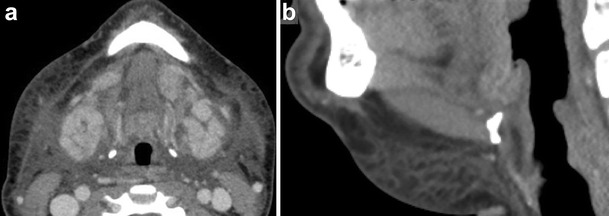
Ludwig angina. **a** Axial and **b** sagittal contrast-enhanced CT images show diffuse inflammatory changes with bands of fluid within the submandibular and sublingual spaces. These changes extend posteriorly into bilateral parapharyngeal spaces, pharyngeal mucosa, and inferiorly along the anterior neck (**b**)

A life-threatening condition, Ludwig angina presents as an intense, rapidly progressive cellulitis, typically originating in the sublingual and submandibular spaces, driven by odontogenic streptococcal infection; immunocompromised patients are more susceptible [13]. Imaging plays a role in identifying airway patency, drainable collections and evidence of gas-forming bacteria. Allowing for rapid evaluation, CT is preferred, which will show diffuse inflammatory changes and bands of fluid, representative of serosanguinous accumulations [19]. As a multispatial process, extension into the parapharyngeal fat/space increases the likelihood of pharyngeal involvement with significant increased risk of impending airway collapse and mediastinitis (Fig. 9). Clinical management involves prompt airway protection, intravenous antibiotic administration and surgical drainage [20].

### Sialadenitis and sialolithiasis

**Fig. 10:**
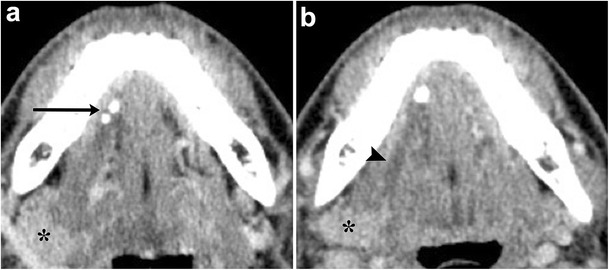
Sialolithiasis with resultant sialadenitis. Axial contrast-enhanced CT images at the level of the submandibular gland demonstrate two well-circumscribed calcifications in the distal right submandibular duct (**a**, *arrow*). The right submandibular duct is dilated proximal to the stones (**b**, *arrowhead*). There is enlargement and enhancement of the right submandibular gland (*) compared to the normal left submandibular gland

Salivary stones (sialoliths) represent calcium concretions, most commonly within the submandibular ductal system, owing to the relatively higher calcium and phosphate salt concentrations of the gland’s secretions [21]. The resultant inflammation clinically manifests as glandular tenderness and swelling, particularly after meals. Sialadenitis is best evaluated with CT, which will show increased size and density of the gland, with ductal calcifications; post-contrast imaging will show diffuse, intense enhancement (Fig. 10) [2]. Advanced stages of sialadenitis can present with suppurative infections and abscess formation [22, 23]. The differential diagnosis of abscess within the salivary glands includes superinfected cysts in HIV patients, suppurative and necrotic lymph nodes, and cystic degeneration of malignancy; therefore, clinical context must be reviewed carefully, and follow-up to resolution may be warranted [22].

**Fig. 11:**
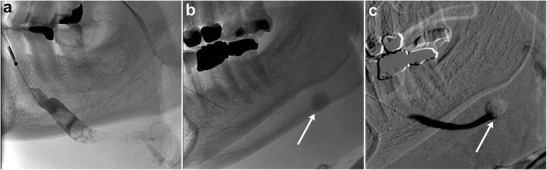
Sialolithiasis. **a** Conventional sialogram demonstrates multiple filling defects within Wharton’s (submandibular) duct, consistent with sialoliths. **b**, **c** In another patient, pre- and post-contrast injection sialogram images demonstrate duct obstruction at the site of a large stone (no contrast is present proximal to the stone, *see arrow*)

Ductal obstruction and calculi as small as 3 mm can be detected on ultrasound, although it is operator dependent [24]. Sialography is the reference standard imaging modality, as it can demonstrate ductal obstruction from multiple aetiologies, including recurrent infection, autoimmune processes and trauma (Fig. 11). MRI and conventional digital subtraction sialography have been found to be comparable in diagnostic performance [25].

## Vascular

Although vascular malformations commonly occur in the head and neck, they are rare within the sublingual and submandibular spaces. Congenital in nature, these lesions are present at birth, but are discovered later in life as they grow with the patient. They are classified based on the primary constituent channel and rate of flow, with resulting distinctive imaging characteristics (Table 3). Slow-flow lesions consist of lymphatic, venous and venolymphatic networks, while high-flow lesions exploit an arteriovenous communication. Initial evaluation may be performed with ultrasound; however, complete characterisation requires CT or MRI [26].

**Table 3:** Distinguishing imaging features among vascular malformations of the sublingual and submandibular spaces

	Lymphatic	Venous	Arteriovenous
CT	-Uniloculated or multiloculated cyst with variably enhancing septa	-Heterogeneous, enhancing infiltrative lesion -Phlebolith(s) are nearly pathognomic if present	-Enlarged artery and vein with shunting
MR	-Uniloculated or multiloculated lesions with high T2 signal -Fluid-fluid level may be present	-Ill-defined lesions with variable T2 signal depending on venous vessel calibre -Small = more solid with lower T2 signal -Large = flow voids	-Serpiginous flow voids representative of arteries and draining veins
US	-Variable echogenicity depending on size of cystic components	-Mixed echogenicity components -Slow monophasic venous flow	-Enlarged feeding artery and dilated veins with arterialised flow -Stacked, biphasic waveform
Site	-Submandibular space -Posterior cervical space	-Deep neck spaces -Maxilla or mandible	-Rare in this region -Usually involves lingual artery

### Low-flow lesions

Characterised by slow vascular flow, this category predominantly consists of venous-based lesions or venous malformations, which most frequently involve the floor of the mouth or buccal space when they occur in the head and neck [13]. Histologically, they are composed of a mass-like collection of venous sinusoids. On initial evaluation, typically by ultrasound, they appear as compressible mixed echogenicity structures with ill-defined margins and monophasic low velocity flow on Doppler interrogation. CT and MRI are used to evaluate spatial extent, visceral involvement and osseous destruction [27]. On MRI, signal patterns depend on vessel size and range from hyperintense venous lakes to more solid appearing lesions that are isointense to muscle on T2-weighted imaging. CT attenuation follows a similar pattern [13]. Characteristic features of any venous-based anomaly, such as phleboliths or diffuse venous phase enhancement, are also present [1].

Infantile haemangiomas are vascular tumours, rather than malformations, as they exhibit a distinct life cycle: manifest in infancy with initial rapid growth and proliferation, followed by involution in early childhood. Clinical presentation is usually sufficient in diagnosis without imaging assistance [1].

Contributing to the slow flow spectrum, lymphatic malformations, also known as lymphangiomas, are cystic structures with partial enhancement and more commonly occur in the submandibular space. Characteristic appearance is that of a non-enhancing multiloculated cystic lesion with fluid-fluid levels without phleboliths [18]. Unilocular lesions are less common and may be mistaken for a thyroglossal duct cyst or duplication cyst [10].

**Fig. 12:**
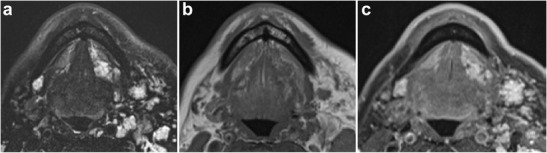
Mixed veno-lymphatic malformation. **a** Axial T2-weighted MR image shows multiple trans-spatial high-signal lesions scattered predominantly throughout the left neck spaces, including the left sublingual space. **b**, **c** Pre- and post-contrast T1-weighted images show enhancement of these lesions

Mixed veno-lymphatic lesions share imaging features of both venous and lymphatic malformations, namely high signal on T2-weighted imaging attributed to the lymphatic component and homogenous enhancement attributed to the venous component (Fig. 12) [10].

### High-flow lesions

Arteriovenous malformations (AVMs) may present at any age and largely involve the lingual artery and vein when they occur in the sublingual space. The lesion is characterised on imaging by enlarged, tortuous vessels consisting of a prominent feeding artery and a draining vein. Occasionally, these lesions may exhibit intraosseous extension [13].

## Benign neoplasms

### Lipoma

Lipomas are the most common type of mesenchymal tumour; however, they rarely arise within the oral cavity. They are encapsulated mature adipose tissue and can be distinguished from surrounding fat based on the internal architecture, forming a well-circumscribed predominantly fat-containing lesion with fibrous septa on CT and MRI [18]. Lack of calcifications and cystic components distinguishes these lesions from dermoid cysts [13].

### Pleomorphic adenoma

**Fig. 13:**
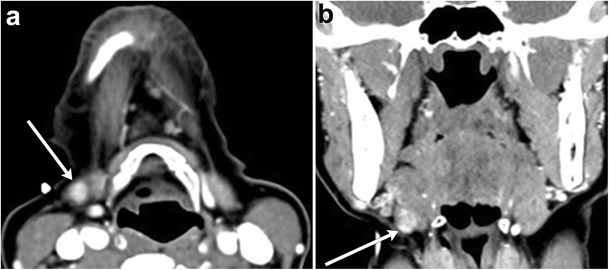
Pleomorphic adenoma of the submandibular gland. **a** Axial and **b** coronal contrast-enhanced CT images demonstrate an enhancing well-circumscribed round mass within the inferior aspect of the right submandibular gland (*arrow*)

Pleomorphic adenomas are the most common benign primary lesions of the salivary glands, and occur in the parotid, sublingual and submandibular glands, in order of decreasing prevalence [15]. These tumours arise from salivary gland rests and are composed of epithelial, myoepithelial and stromal components. On CT, they usually appear as well-circumscribed homogenously enhancing masses, which demonstrate high signal on T2-weighted imaging (Fig. 13). As the lesion enlarges, areas of haemorrhage and cystic necrotic changes may develop, thus altering the classic imaging appearance [13]. Rapid growth should raise suspicion for malignant degeneration, which occurs in about 15% of cases [1]. Early changes are better delineated on MRI, with loss of homogenous high signal on T2-weighted images or rupture of the fibrous capsule [22].

### Nerve sheath tumours

Schwannomas and neurofibromas are rare within the sublingual and submandibular spaces. Arising from peripheral and sympathetic nerves, schwannomas involving the lingual and hypoglossal nerves can occur in the floor of the mouth. Neurofibromas are associated with known neurofibromatosis. On CT, these lesions are described as enhancing homogenous isoattenuating masses with distinct margins. On MRI, they demonstrate isointense signal on T1-weighted imaging and hyperintense signal on T2-weighted imaging, relative to the adjacent musculature. Larger lesions can have cystic components [13].

## Malignant neoplasms

**Fig. 14:**
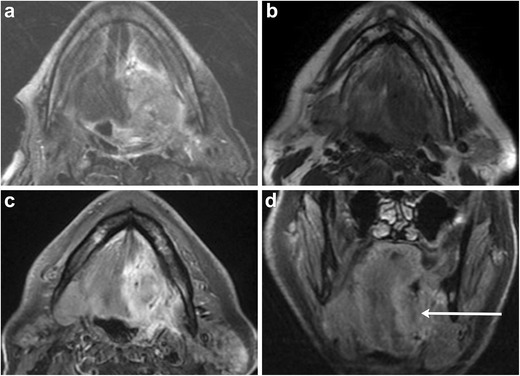
Squamous cell carcinoma involving the left sublingual space. Infiltrative lesion centred within the left sublingual space, which demonstrates heterogenous intermediate to high signal on T2-weighted MRI (**a**) and isointensity compared with adjacent musculature on T1-weighted MRI (**b**). **c** Post-contrast axial and **d** coronal T1-weighted MR images reveal heterogenous enhancement (*arrow*)

**Fig. 15:**
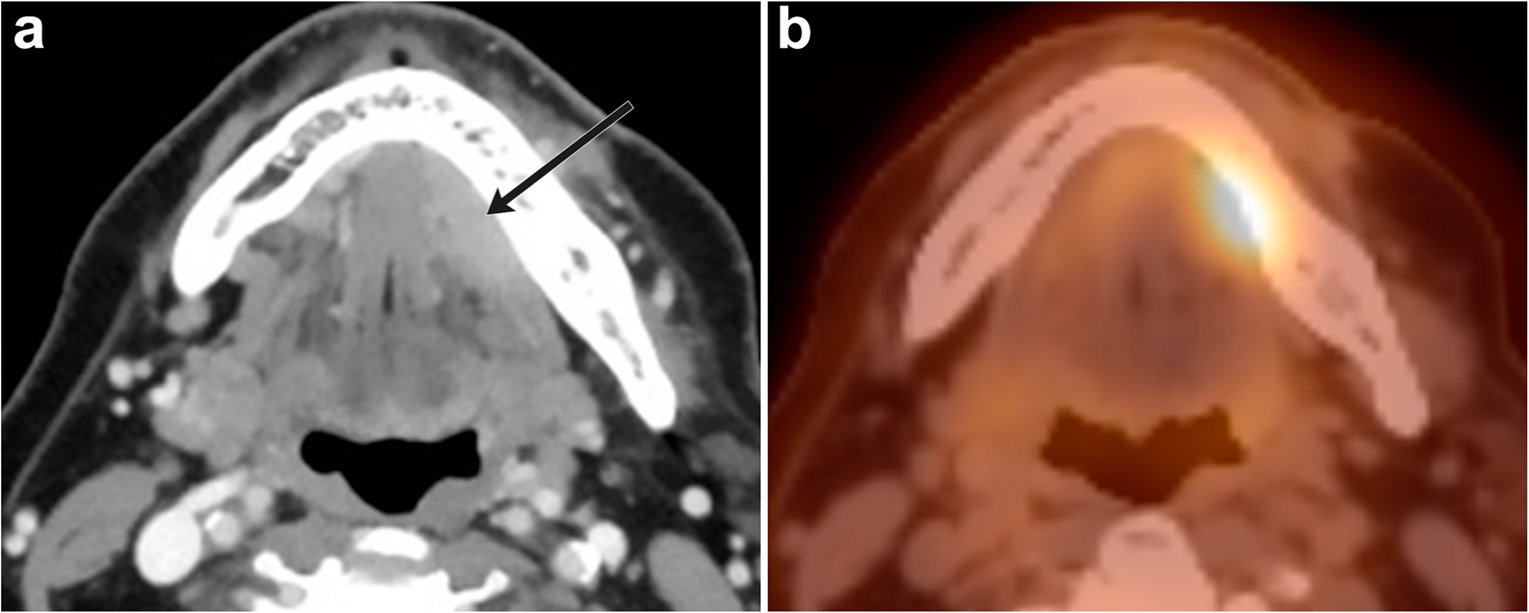
Squamous cell carcinoma involving the left sublingual space. **a** Axial contrast-enhanced CT image shows a homogenously enhancing lesion within the left sublingual space (*arrow*), **b** which demonstrates ^18^F-fluorodeoxyglucose (FDG) avidity on PET/CT

**Fig. 16:**
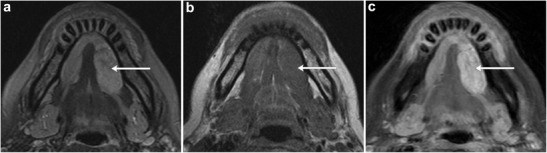
Adenoid cystic carcinoma of the sublingual gland. **a** Axial T2-weighted and **b** T1-weighted MR images of the sublingual space demonstrate a lobulated high T2 signal and low T1 signal lesion enlarging and distorting the left sublingual gland (*arrow*). **c** There is diffuse enhancement on the axial post-contrast T1-weighted MR image

Malignant lesions within this region typically represent invasive squamous cell carcinoma originating in the mucosa of the floor of the mouth or the relatively rare primary salivary gland neoplasms, most commonly adenoid cystic carcinoma in submandibular gland and mucoepidermoid carcinoma in the parotid gland. Primarily evaluated on CT and MRI, these malignant processes are often indistinguishable on imaging, resembling infiltrative lesions with similar routes of spread including perineural and osseous extension (Figs. 14, 15, and 16) [18, 28]. MRI helps in assessment of tumour extent, specifically defining depth of invasion, a critical component in the T-staging of oral cavity cancer [13, 29, 30].

Lymphoma of the salivary glands is rare, accounting for about 5% of non-Hodgkin’s lymphomas with mucosa-associated lymphoid tissue (MALT) lymphoma as the most common subtype. Salivary glands may acquire lymphocytes due to chronic inflammation or autoimmune disorder, such as Sjogren’s syndrome or arise from intra-parotid lymph nodes. Imaging features typically mimic those of other lymphoproliferative disorders with homogeneously enhancing nodules on CT, which demonstrate homogeneous intermediate signal on all MRI sequences [22, 31].

## Pseudo-lesion

### Mylohyoid defects with herniation of sublingual space contents

**Fig. 17:**
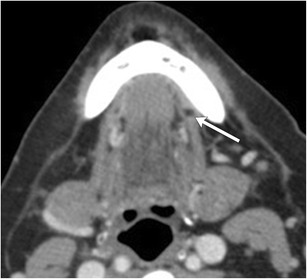
Mylohyoid slit (boutonniere). Axial contrast-enhanced CT image shows a linear, fat-containing defect within the mylohyoid muscle at the anterior aspect (*arrow*)

**Fig. 18:**
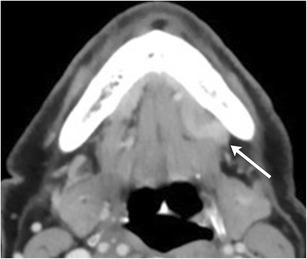
Sublingual gland herniation, mimicking a palpable “lesion” on physical exam. Axial contrast-enhanced CT image demonstrates herniation of the left submandibular gland through a mylohyoid muscle sling defect

The mylohyoid muscle is traditionally depicted as a continuous muscular sling formed from two halves that join at the fibrous median raphe. However, with modern day CT and MRI, focal defects are becoming increasingly evident and have been reported in up to 77% of CT studies. During embryological development, the anterior and posterior muscular sheets fail to overlap with a resultant slit, allowing for protrusion of the sublingual space contents into the submandibular space (Fig. 17). Also known as the mylohyoid boutonniere, the protruding salivary gland tissue, fat, blood vessels or a combination of the three can present as a palpable “mass” on physical exam (Fig. 18) [32].

### Sialosis

Symmetrical, diffuse and painless enlargement of the sublingual and submandibular glands may reflect response to a systemic process, such as diabetes mellitus, hypothyroidism, chronic alcoholism, malnutrition and certain drugs such as antibiotics, diuretics and antipsychotics. Bilateral and symmetric involvement should clue one into the possibility of a systemic diagnosis [22].

## Conclusions

As seen, a variety of pathology occurs within the sublingual and submandibular spaces, as well as the surrounding regions. Utilising knowledge of embryological origin of the tissues, anatomical relationships and characteristic imaging features can help the radiologist in accurately diagnosing pathology.
